# Supplementary material for: Comparison of Methods for Incorporating Related Data When Developing Clinical Prediction Models: A Simulation Study
Source: Biom J. 2026 Aug 27;68(5):e70166. doi: 10.1002/bimj.70166 (PMC13520826; doi:10.1002/bimj.70166)
Supplement: Supplementary file 2 [file BIMJ-68-e70166-s001.zip › third submission code review - R2/reproducibility_supplement/results/Plots/Individual_plots/indv_stability_plot_intercept_calibration_source_only_model15_combination_SC_ID8_TS0.25_DS4.pdf]

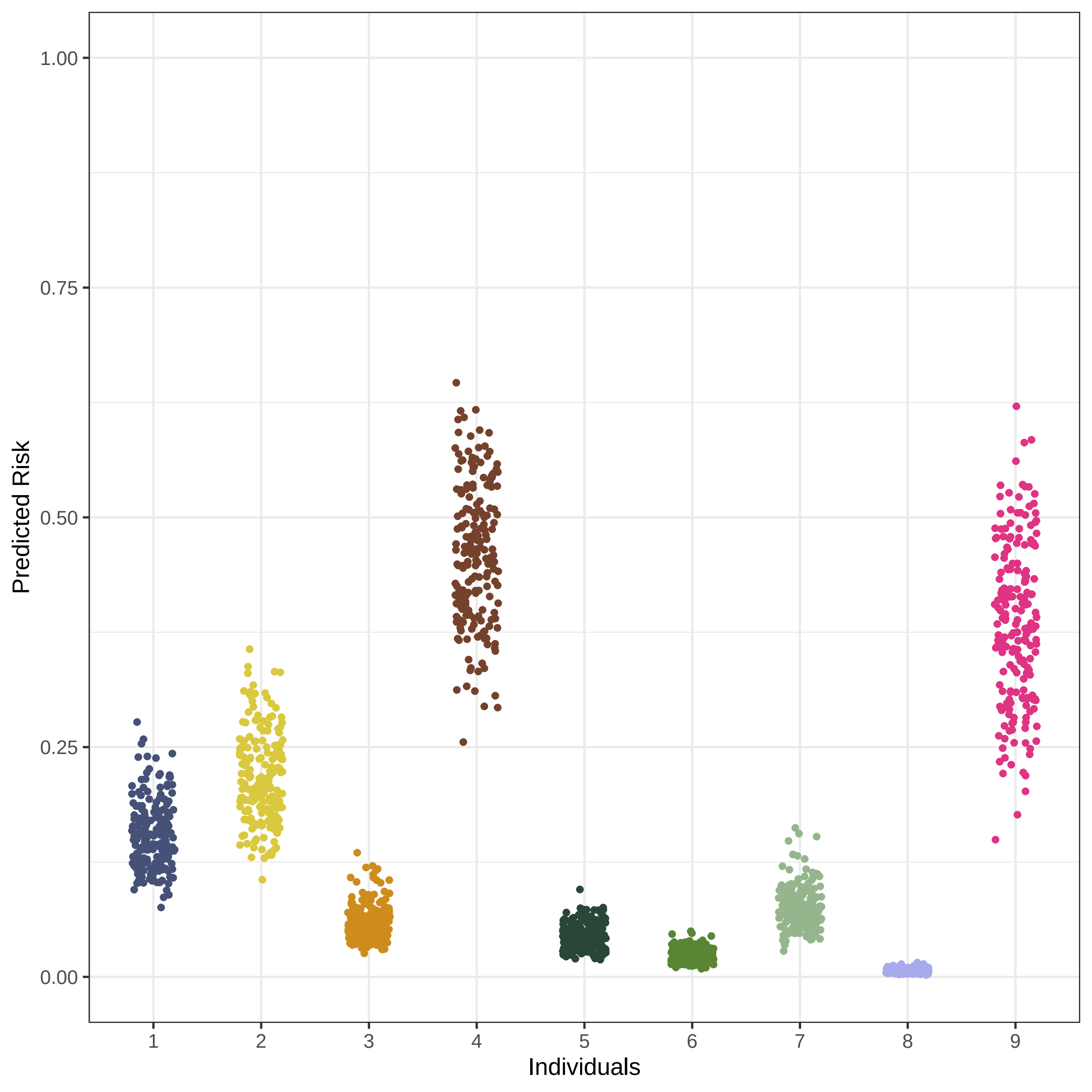

1.00
0.75
0.50
0.25
0.00
Predicted Risk
1
2
3
4
5
6
7
8
9
Individuals
